# Field-Compatible Cytometric Assessment of Epididymal Alpaca Sperm Viability and Acrosomal Integrity Using Fluorochrome

**DOI:** 10.3390/ani15152282

**Published:** 2025-08-05

**Authors:** Alexei Santiani, Miguel Cucho, Josselyn Delgado, Javier Juárez, Luis Ruiz, Shirley Evangelista-Vargas

**Affiliations:** 1 Laboratory of Animal Reproduction, Faculty of Veterinary Medicine, Universidad Nacional Mayor de San Marcos (UNMSM), 2800 Circunvalación Av., San Borja, Lima 15021, Peru05080008@unmsm.edu.pe (J.J.); lruizg@unmsm.edu.pe (L.R.); 2 Grupo de Investigación en Biotecnología Reproductiva Animal, Dirección General de Investigación, Desarrollo e Innovación, Universidad Científica del Sur (UCSUR), Panamericana Sur Km. 19, Villa el Salvador, Lima 15018, Peru; sevangelista@cientifica.edu.pe

**Keywords:** alpaca, spermatozoa, field-based evaluation, flow cytometry, viability, acrosomal integrity, reproductive management

## Abstract

In remote alpaca breeding regions, access to advanced sperm analysis laboratories is limited. This study validates a practical cytometric method for evaluating sperm viability and acrosomal integrity in epididymal alpaca sperm using early fluorochrome staining, formaldehyde fixation, and intermediate storage. Thirty-two testes were transported at 5 °C, and spermatozoa were collected from the cauda epididymis. After morphometric screening, 26 samples were included. Aliquots were stained with Zombie Green (viability) and FITC–PSA (acrosomal integrity), at time zero. Each aliquot was divided for cytometric analysis at T0 (immediately), T24 (24 h after formaldehyde fixation) and T1w (1 week post-fixation). Fixed samples showed higher viability and acrosomal integrity values (T24: 70.75%, 97.24%; T1w: 71.80%, 97.21%) than T0 (67.63%, 95.89%). This may reflect fluorescence alterations associated with fixation. Strong correlations and Bland–Altman analysis confirmed consistency across time points. This method enables accurate sperm quality evaluation up to one week after collection, offering a useful tool for reproductive monitoring in field conditions without immediate analysis. Further research on ejaculated semen and field protocols is recommended.

## Simple Summary

Alpacas are important animals in the Andes, especially for rural families who rely on them for income. Ensuring good reproductive performance in males is essential, but evaluating sperm quality often requires advanced laboratory tools that are not available in remote areas. This study offers a simple and practical method to assess the quality of alpaca sperm collected after death, using two special dyes that identify whether the sperm cells are alive and whether they are structurally intact. After staining, the samples were preserved in a fixative and stored at 5 °C for up to one week before being analyzed. The results showed that the method is reliable, with consistent findings even after storage. This approach is helpful for veterinarians and researchers who work in the field and need a way to collect and preserve sperm samples until they can be analyzed in a laboratory. While this study used sperm from the epididymis, future research should focus on ejaculated semen. Overall, this method can support better reproductive management and genetic conservation programs in alpacas, especially in rural regions where access to equipment is limited but fertility monitoring remains important.

## 1. Introduction

Flow cytometry enables objective, multi-parameter analysis of spermatozoa, evaluating over 10,000 cells in seconds [1], and is well established for assessing sperm viability, mitochondrial membrane potential, acrosomal integrity, DNA fragmentation, and apoptosis [2]. In alpacas, the evaluation of sperm parameters has been progressively standardized through various studies. For ejaculated spermatozoa, viability, acrosomal integrity, DNA integrity, and mitochondrial membrane potential have been characterized [3]. In epididymal sperm, additional work has addressed acrosomal integrity, lipid peroxidation, and mitochondrial function [4], as well as apoptotic markers [5]. However, its routine application in veterinary field settings remains limited, especially in remote breeding centers where laboratory infrastructure is scarce and rapid transport of fresh samples is often unfeasible. As a result, fertility assessment in these contexts is often restricted to basic parameters such as motility.

Most existing methods for sperm evaluation require viable cells, restricting their applicability under field conditions. A technique has been proposed for assessing stallion sperm using markers that remain bound to cells after formaldehyde fixation, including Zombie Green, an amine-reactive fluorochrome for identifying dead cells, and MitoTracker Deep Red for assessing mitochondrial activity [6]. Amine-reactive fluorochromes irreversibly label dead cells even after formaldehyde fixation, allowing identification by flow cytometry [7,8]

The use of MitoTracker fluorochromes to estimate the subpopulation of spermatozoa with mitochondrial activity [9], with the ability to emit fluorescence even after formaldehyde fixation [10], has been reported in alpacas [4,11]. A strong correlation (r = 0.88) has been reported between sperm viability and mitochondrial membrane potential assessed by MitoTracker, suggesting that evaluating sperm viability alone could provide sufficient information without the need for separate mitochondrial membrane potential analysis [11]. Additionally, FITC–PSA has been used to assess acrosomal integrity in alpaca sperm [12], but the effect of fixation on its fluorescence emission in alpaca spermatozoa has not yet been explored.

Although formaldehyde has been used for decades to preserve sperm for morphological or fertilization studies [13], the objective of the present study is not to develop a new preservation protocol. Rather, it seeks to assess whether early staining with Zombie Green and FITC–PSA followed by fixation can allow intermediate-term evaluation of sperm viability and acrosomal integrity by flow cytometry in epididymal alpaca sperm. The focus is not on preserving fertilizing capacity, but on enabling functional sperm assessment when immediate analysis is not possible—such as in field-based breeding programs. In this context, intermediate-term evaluation refers to a practical time window of up to one week after collection and fixation, during which samples can be stored under refrigeration before cytometric analysis. This aligns with realistic scenarios in remote areas, where cytometers may not be immediately available.

If the use of these fluorochromes and the effect of fixation on their fluorescence emission can be validated, as preliminary results suggest, flow cytometry could become a more accessible method for evaluating sperm viability and acrosomal integrity in alpaca breeding centers, thus improving fertility assessment under field conditions. However, further validation is necessary, particularly with semen samples and field application, before widespread adoption can be recommended. This study aimed to conduct a preliminary validation of this practical technique for evaluating sperm viability and acrosomal integrity in epididymal alpaca sperm processed under field conditions.

## 2. Materials and Methods

### 2.1. Location

Alpaca testicles/epididymides were obtained from the Municipal Slaughterhouse of Ninacaca, Pasco, Peru, located at an altitude of 4100 m a.s.l. Sperm processing was carried out in a standard laboratory environment at sea level (FMV–UNMSM, Lima, Peru).

### 2.2. Sperm Samples and Retrieval

Thirty-two alpaca testicles/epididymides were collected, generally in pairs from individual males. However, only one testicle per male was selected to ensure the independence of experimental units. Selection was based on morphometric criteria, with preference given to the larger testicle (by weight and length). Testicles were collected between 10:00 and 12:00 h, immediately after slaughter. They were rinsed with 0.9% sodium chloride solution, placed in plastic bags containing the same solution at 5 °C, and packed in a Styrofoam box with refrigerant gel to maintain the temperature during transport, as described previously [4]. Samples were shipped via interprovincial bus service departing in the late afternoon or evening, and arrived in Lima between 06:00 and 08:00 h the following morning. Upon arrival at the laboratory, testicles were immediately processed. The total elapsed time from collection to processing was approximately 20 h.

Twenty-six testicles meeting the inclusion criteria (>10 g in weight and >3 cm in length) were selected, each corresponding to a different male, and processed to confirm the presence of spermatozoa, following established protocols [14]. In the laboratory, the caudal region of the epididymis was separated and placed in a Petri dish heated to 37.5 °C. Then, 1 mL of PBS was added to facilitate sperm release through serial cuts. The resulting suspension was collected in a 1.5 mL Eppendorf tube and kept at 37.5 °C. Sperm concentration and motility were evaluated using a phase-contrast microscope (400×).

### 2.3. Experimental Design and Sample Processing

This study involved two single staining protocols, with sperm samples evaluated at three time points: (1) T0: fresh epididymal spermatozoa evaluated immediately after recovery (0 h); (2) T24: spermatozoa fixed with formaldehyde and stored for 24 h before analysis; and (3) T1w: spermatozoa fixed with formaldehyde and stored for 1 week before analysis.

Each sample (n = 26) was divided into six 100 µL aliquots to assess sperm viability and acrosomal integrity separately. For each fluorochrome, stained aliquots were analyzed at three time points: immediately after staining (T0), after 24 h of fixation and refrigerated storage (T24), and after 1 week under the same conditions (T1w). All fixed samples were stored at 4 °C in the dark until flow cytometric analysis.

### 2.4. Sperm Viability Assessment (Using Zombie Green)

The procedure for fixed sperm evaluation was conducted according to established protocols using Zombie Green fluorochrome (#423111, Biolegend, San Diego, CA, USA) [6]. For this purpose, 100 μL of each sample was incubated with 1 μL of a working solution prepared by diluting the stock 1:100 in DMSO. The samples were incubated at 22 °C for 30 min in the dark. After incubation, the fluorochrome was removed by washing with 1 mL of PBS and centrifugation at 600× *g* for 8 min. The subsequent step depended on the time point: samples corresponding to T0 were resuspended in 100 µL of PBS and immediately analyzed by flow cytometry (unfixed). In contrast, samples assigned to T24 or T1w were resuspended in 100 µL of 2% formaldehyde to initiate fixation, then stored at 4 °C in the dark until analysis. Sperm exhibiting intense green fluorescence (Zombie Green-positive) were considered non-viable (dead), while sperm showing minimal or no fluorescence (Zombie Green-negative) were considered viable. Viability results were expressed as the percentage of viable sperm.

### 2.5. Acrosome Integrity Assessment (Using FITC–PSA)

Each sperm suspension (100 μL) was incubated with 2.5 μL of FITC–PSA (Sigma-Aldrich, St. Louis, MO, USA; Stock concentration: 100 μg/mL) to achieve a final concentration of 2.5 μg/mL, following the protocol previously established for acrosomal assessment [4]. Samples were incubated at 38 °C for 10 min in the dark. Unfixed samples were analyzed immediately by flow cytometry without washing. For fixed samples, the subsequent fixation, storage, washing, and delayed analysis were performed following the protocol described in the next subsection. FITC–PSA binds specifically to the acrosomal region; spermatozoa exhibiting green fluorescence in this region (FITC–PSA-positive) were considered to have acrosomal damage, while those without detectable fluorescence (FITC–PSA-negative) were considered to have intact acrosomes. The results were expressed as the percentage of spermatozoa with intact acrosomes.

### 2.6. Sperm Fixation

After staining with the corresponding fluorochromes, only samples destined for later time points (T24 and T1w) were fixed using formaldehyde solutions. For Zombie Green-stained samples, the pellet was resuspended in 100 µL of 2% formaldehyde. For FITC–PSA-stained samples, 100 µL of 4% formaldehyde was added to 100 µL of sperm suspension to achieve a final concentration of 2%. All fixed samples were stored at 4 °C in the dark, and analyzed after 24 h (T24) or after 1 week (T1w). Before analysis, samples were washed twice by resuspending the pellet in phosphate-buffered saline (PBS) followed by centrifugation at 600× *g* for 8 min to remove residual fixative.

### 2.7. Flow Cytometry

**Figure 1:**
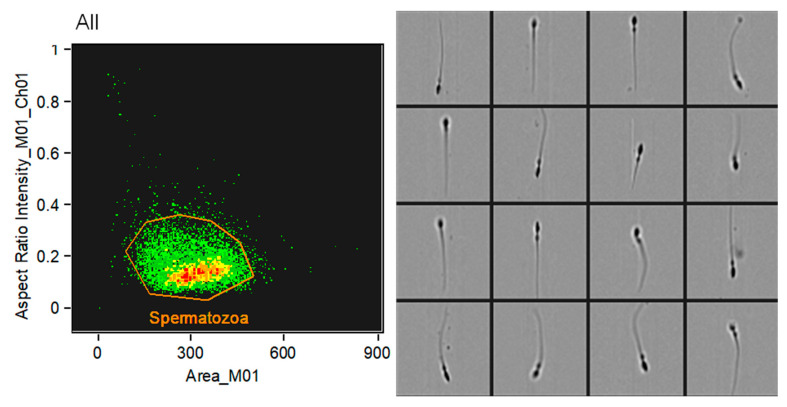
Identification of the sperm population using imaging flow cytometry. On the left, a dot plot illustrates the selection of spermatozoa based on morphological parameters: area (*x*-axis) and aspect ratio (*y*-axis). This gating strategy allowed the discrimination of the sperm population from debris and other non-sperm events. On the right, representative images of events within the gated population confirm their spermatozoa identity, validating the selection criteria based on morphological features.

Samples were analyzed using a FlowSight imaging flow cytometer (Amnis Corporation, Seattle, WA, USA), equipped with an integrated image-based analysis system. Data acquisition was carried out using the INSPIRE software (v. 100.3.218.0), and subsequent analyses were performed with the IDEAS software (v. 6.2; Amnis Corporation, Seattle, WA, USA). For each sample, 10,000 events compatible with spermatozoa were acquired based on morphological parameters, specifically, the cell area and aspect ratio (length-to-width relationship). Image verification was performed to confirm that the selected events corresponded to individual sperm cells, allowing for accurate gating of distinct subpopulations (Figure 1).

**Figure 2:**
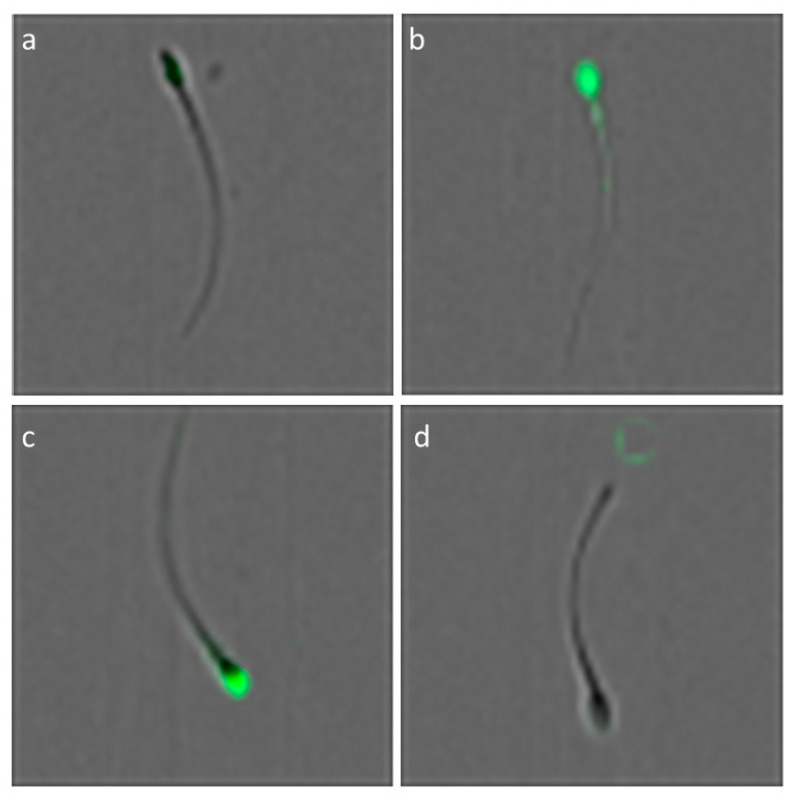
Representative image of an alpaca spermatozoa obtained by imaging flow cytometry: (**a**) Zombie Green-negative (viable), (**b**) Zombie Green-positive (non-viable), (**c**) FITC–PSA-positive (reacted/damaged acrosome), and (**d**) FITC–PSA-negative (intact acrosome).

To ensure the specificity of fluorescent signal detection, autofluorescence controls were included during setup, and each fluorochrome was analyzed independently to avoid spectral overlap. Zombie Green and FITC–PSA were excited using a 488 nm laser, with fluorescence emission collected through the 505–560 nm channel (Channel 02). Image-based analysis allowed validation of fluorescence localization, confirming that signal patterns corresponded to expected viability and acrosomal integrity profiles. Representative images confirming the localization patterns of Zombie Green and FITC–PSA fluorescence in individual spermatozoa are shown in Figure 2.

### 2.8. Statistical Analysis

A repeated measures ANOVA was performed to compare time points (T0: 0 h unfixed vs. T24: 24 h and T1w: 1 week fixed in formaldehyde). When significant differences were detected, Tukey’s multiple comparison test was applied as a post hoc analysis to identify specific group differences. Differences were considered statistically significant when *p* < 0.05. Results were expressed as mean ± standard deviation. Pearson’s correlation (r) was used to assess the relationship between the percentage of sperm viability and the percentage of acrosomal integrity between the time point T0 (0 h) and T24 (24 h) and T1w (1 week). Bland–Altman plots were used to visualize the differences between paired measurements of the same samples. The mean of the differences was used to estimate the mean bias between time points, and the limits of agreement were calculated to assess the degree of concordance.

## 3. Results

A total of 26 epididymal sperm samples met the inclusion criteria and were processed. The corresponding testicles had a mean length of 3.6 ± 0.4 cm and a mean weight of 14.41 ± 3.28 g. Sperm concentration averaged 122.54 ± 102.50 million/mL, and the mean total motility was 51.15 ± 15.96%. These baseline parameters confirmed the suitability of the samples for evaluating the viability and acrosomal integrity of epididymal spermatozoa using fluorochrome-based cytometric analysis. The raw data used for this analysis are provided in the Supplementary Materials.

**Figure 3:**
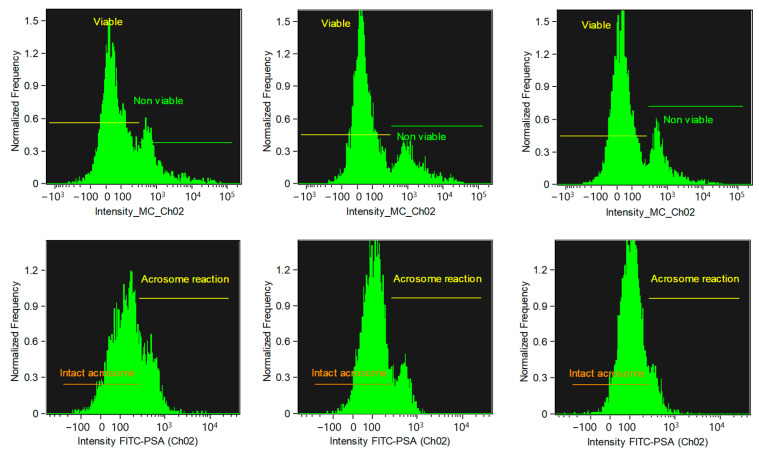
Histograms of alpaca spermatozoa incubated with Zombie Green (top row) and FITC–PSA (bottom row). The left column shows unfixed samples at T0, the middle column shows samples fixed and stored for 24 h (T24), and the right column shows samples fixed and stored for one week (T1w). The proportion of non-viable spermatozoa (**top**) and spermatozoa with reacted acrosomes (**bottom**) remains similar across all evaluated time points.

When comparing the sperm viability percentages in alpacas, samples analyzed immediately after collection (T0, unfixed) showed significantly lower values (*p* < 0.01) compared to those fixed and stored for 24 h (T24) or 1 week (T1w). No significant differences were observed between T24 and T1w (*p* > 0.05). Similarly, when comparing the acrosomal integrity percentages, values at T0 were significantly lower (*p* < 0.01) than both T24 and T1w, with no significant differences observed between the two fixed groups (*p* > 0.05) (Table 1 and Figure 3).

Correlation analysis between viability at T0 and at T24 showed a strong positive correlation, which remained similarly strong between T0 and T1w. Likewise, the correlation between acrosomal integrity at T0 and acrosomal integrity at T24, as well as between acrosomal integrity at T0 and T1w, were all significant, positive, and strong (Table 2).

**Figure 4:**
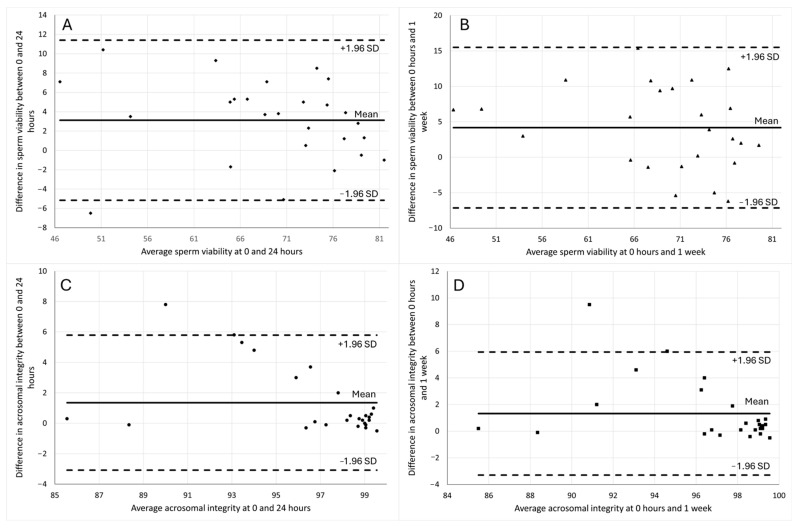
Bland–Altman plots assessing sperm viability and acrosomal integrity at different time points. (**A**) Comparison of sperm viability at T0 (0 h) vs. T24 (24 h), (**B**) comparison of sperm viability at T0 (0 h) vs. T1w (1 week), (**C**) comparison of acrosomal integrity at T0 (0 h) vs. T24 (24 h), and (**D**) comparison of acrosomal integrity at T0 (0 h) vs. T1w (1 week).

In the analysis of the Bland–Altman plots (Figure 4), the agreement between measurements of sperm viability and acrosomal integrity at different time points (T0 vs. T24 and T0 vs. T1w) was assessed. For sperm viability, the mean difference between T0 and T24 (Figure 4A) was 3.12 ± 4.22%, and between T0 and T1w (Figure 4B) was 4.17 ± 5.78%. For acrosomal integrity, the mean difference between T0 and T24 (Figure 4C) was 1.35 ± 2.26%, and between T0 and T1w (Figure 4D) was 1.32 ± 2.36%.

In the comparisons of sperm viability and acrosomal integrity at T0 vs. T24 and T0 vs. T1w, only one data point in each of the comparisons of sperm viability at T0 vs. T24 (Figure 4A), acrosomal integrity at T0 vs. T24 (Figure 4C), and acrosomal integrity at T0 vs. T1w (Figure 4D) fell outside the limits of agreement, indicating slight discrepancies in these specific cases. In contrast, for sperm viability at T0 vs. T1w (Figure 4B), all data points fell within the limits of agreement, suggesting strong concordance between the measurements over time.

## 4. Discussion

This study is the first to evaluate alpaca epididymal spermatozoa using Zombie Green and FITC–PSA fluorochromes to assess sperm viability and acrosomal damage, respectively, in fixed samples. It provides a novel approach by comparing freshly obtained spermatozoa (T0) with samples fixed in formaldehyde and stored under refrigeration for up to one week (T24 and T1w). While previous studies have explored several aspects of alpaca sperm biology, none have specifically addressed the use of these fluorochromes in fixed samples [4,5,11,12,15,16]. Therefore, this study contributes a methodological advancement with potential applicability in both research- and field-based reproductive assessments.

Zombie Green has been primarily used to assess sperm viability in stallions [6,17], and it has also shown versatility in labeling other cell types, including murine [18], human [19], and protozoan cells [20]. Our results suggest that sperm viability can be evaluated in formaldehyde-fixed samples even after one week of storage, demonstrating the potential of Zombie Green as a viable tool for delayed analysis. This is particularly relevant in alpacas, where field collection often occurs in remote areas and immediate laboratory access is not always feasible.

Similarly, FITC–PSA has been extensively used to assess acrosomal integrity in fresh alpaca sperm samples [3,4,12,16]. Our findings extend its application by demonstrating that FITC–PSA can reliably detect acrosomal damage in fixed sperm samples analyzed via flow cytometry. This method could be especially advantageous for field veterinarians and researchers working under logistical constraints, allowing them to fix samples on-site and conduct detailed evaluations later in laboratory settings without compromising diagnostic quality. However, a limitation of our protocol is the absence of a washing step after FITC–PSA staining in fixed samples. While this approach simplified sample handling under field-simulated conditions, prolonged exposure to the staining solution may lead to overstaining or background artifacts.

Although all staining, incubation, and fixation procedures in this study were conducted under laboratory conditions, these steps can feasibly be carried out in the field using a portable setup. The required materials include basic pipetting tools, fluorochromes, a water bath or portable incubator, a low-speed centrifuge, and a refrigeration source. While we did not implement this protocol in the field, our results suggest that on-site staining and fixation are realistic under field conditions, with subsequent analysis by flow cytometry performed in the laboratory. This distinction has been clarified to emphasize that our current findings support potential field implementation, though further validation under actual field conditions is still needed.

The feasibility of collecting alpaca spermatozoa in the field, processing them on-site by staining and fixation, and storing them under refrigeration for up to one week before cytometric analysis has significant practical implications. In particular, it offers greater flexibility in managing time-sensitive procedures such as sperm viability assessments in rural or underserved regions. Although the term “intermediate-term” is often associated with longer storage durations, we define it here as a practical one-week window, which aligns with realistic field scenarios. However, applying this method in practice requires careful control of storage conditions, especially temperature stability, and proper fixation technique [21]. Environmental factors such as high ambient temperatures, lack of refrigeration, and the absence of sterile supplies may introduce variability in sperm preservation outcomes [22], and future protocols should address these challenges, potentially through portable and user-friendly sperm processing kits.

A repeated measures ANOVA was performed to account for intra-sample correlations [23], revealing statistically significant differences between T0 and the two time points T24 and T1w in terms of both sperm viability and acrosomal integrity. Interestingly, the fixed samples showed higher percentages of viability and acrosomal integrity compared to the fresh, unfixed T0. However, this apparent improvement does not reflect a true biological enhancement, but rather a technical artifact likely due to the masking of cell damage caused by fixation and storage, which reduces fluorescence intensity in compromised sperm. This is a critical consideration, as both Zombie Green and FITC–PSA function as exclusion markers, and lower fluorescence intensity in compromised cells may result in underestimation of non-viable or acrosome-damaged sperm.

Fixation with formaldehyde has been shown to reduce fluorescence intensity, potentially leading to misinterpretation of cytometric data [24,25]. This phenomenon can be attributed to the formation of protein cross-links that obscure epitope accessibility or alter the local chemical environment of the fluorophores [26,27]. Additionally, chemical modifications such as partial fluorophore oxidation or degradation—particularly of FITC—may further reduce signal strength. The introduction of fixation-induced autofluorescence [28,29] may also interfere with signal detection, especially in flow cytometric applications. These variables emphasize the importance of optimizing fixation protocols and using appropriate controls to account for signal attenuation.

One important limitation of this study is the lack of a fixed aliquot analyzed immediately after fixation (i.e., at 0 h). The inclusion of such a time point would have facilitated a clearer distinction between the effects of fixation itself and those attributable to storage duration. However, the current study design was intentionally chosen to simulate realistic field conditions, where freshly collected sperm samples are rarely analyzed on-site and fixation is used primarily to preserve them for later evaluation. Therefore, the unfixed T0 provides a relevant baseline for optimal laboratory conditions, while the fixed T24 and T1w reflect practical application scenarios. Future studies should explicitly include a fixed 0 h group as an additional control, in order to better isolate the effects of fixation from those of storage time.

Correlation analysis revealed strong, positive, and statistically significant correlations (r > 0.8) between the different time points for both sperm viability and acrosomal integrity, indicating high internal consistency in the measurements across time. To further assess agreement, Bland–Altman analysis was conducted, and most data points fell within the limits of agreement. These findings support the reliability of using fixed samples for cytometric evaluation of sperm quality within a one-week window, reinforcing the practicality of this approach in field settings.

The broader implications of this study extend to genetic conservation programs and reproductive management strategies in alpacas. The ability to evaluate sperm quality from fixed samples after field collection enables more objective, time-efficient, and logistically feasible fertility assessments, particularly in conservation breeding of endangered or geographically isolated populations. Unlike current field evaluations—often limited to subjective motility estimates or basic eosin staining—this approach allows for more reliable measurements of sperm viability and acrosomal integrity. Standardizing such methods could not only enhance male selection but also pave the way for validating additional functional assays, such as mitochondrial membrane potential, as proposed for equine sperm [6]. Furthermore, this approach could be adapted for use in other camelids or species with similar logistical constraints in sample collection and analysis. However, it should be noted that this study was conducted exclusively with epididymal spermatozoa, which lack seminal plasma. In the case of ejaculated semen [30], the high viscosity and biochemical properties of alpaca seminal plasma could interfere with fluorochrome penetration and staining efficiency, especially after storage. Therefore, the applicability of this technique to ejaculated sperm requires further validation and protocol adjustments to account for these differences.

## 5. Conclusions

This study demonstrates that Zombie Green and FITC–PSA fluorochromes, combined with formaldehyde fixation, allow reliable evaluation of alpaca epididymal sperm viability and acrosomal integrity up to one week after collection. These findings support the feasibility of using fixed samples for delayed cytometric analysis under field conditions. The method offers a practical tool for reproductive assessments in alpaca breeding programs and lays the groundwork for future adaptation to field scenarios and ejaculated semen.

## Figures and Tables

**Table 1:** Comparison of sperm viability and acrosomal integrity percentages (mean ± standard deviation) in unfixed (0 h) and fixed (24 h and 1 week) alpaca sperm samples. The data represents the differences in cell viability and acrosomal integrity across different time points and sample conditions.

Variables	Groups		
	Unfixed (0 h)	Fixed (24 h)	Fixed (1 week)
Sperm viability (%) (Zombie Green-negative)	67.63 ± 10.20 ^a^	70.75 ± 9.43 ^b^	71.80 ± 8.79 ^b^
Acrosomal integrity (%) (FITC–PSA-negative)	95.89 ± 4.35 ^a^	97.24 ± 3.36 ^b^	97.21 ± 3.49 ^b^

^a, b^ Different letters indicate significant differences (Tukey’s tests; *p* < 0.01) in rows.

**Table 2:** Correlation analysis of sperm viability and acrosomal integrity percentages in unfixed (0 h) and fixed (24 h and 1 week) alpaca sperm samples.

Sperm Parameters	Pearson Correlation		Pairs (n)
	r	*p*	
Viability 0 h vs. viability 24 h	0.9103	*p* ˂ 0.0001	26
Viability 0 h vs. viability 1 week	0.8248	*p* ˂ 0.0001	26
Acrosomal integrity 0 h vs. acrosomal integrity 24 h	0.8589	*p* ˂ 0.0001	26
Acrosomal integrity 0 h vs. acrosomal integrity 1 week	0.8419	*p* ˂ 0.0001	26

## Data Availability

The raw data supporting the findings of this study are available in the Supplementary Materials associated with this publication.

## Supplementary Materials

The following supporting information can be downloaded at: https://www.mdpi.com/article/10.3390/ani15152282/s1, Table S1: Raw values of sperm viability (Zombie Green) and acrosomal integrity (FITC–PSA) in 26 epididymal alpaca sperm samples evaluated at 0 h, 24 h, and 1 week.
